# The enol of isobutyric acid†

**DOI:** 10.1039/d4cc01140f

**Published:** 2024-04-16

**Authors:** Akkad Danho, Artur Mardyukov, Peter R. Schreiner

**Affiliations:** a Institute of Organic Chemistry, Justus Liebig University Heinrich-Buff-Ring 17 Giessen 35392 Germany prs@uni-giessen.de

## Abstract

We present the gas-phase synthesis of 2-methyl-prop-1-ene-1,1-diol, an unreported higher energy tautomer of isobutyric acid. The enol was captured in an argon matrix at 3.5 K, characterized spectroscopically and by DFT computations. The enol rearranges likely photochemically to isobutyric acid and dimethylketene. We also identified propene, likely photochemically formed from dimethylketene.

## Abstract 2

We present the gas-phase synthesis of 2-methyl-prop-1-ene-1,1-diol, an unreported higher energy tautomer of isobutyric acid.

## Graphical abstract

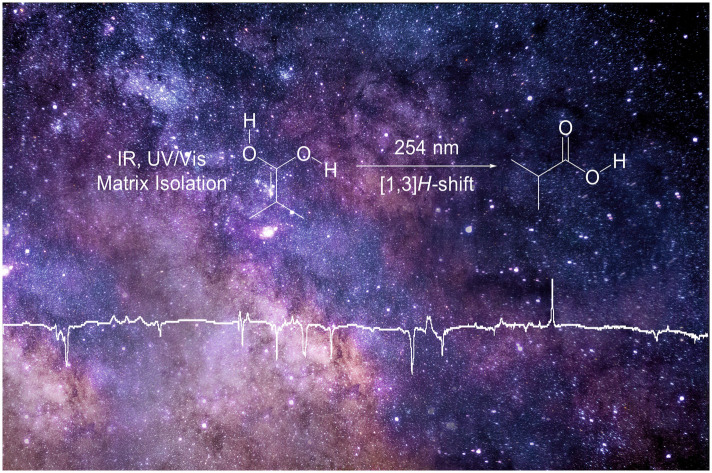

The exploration of interstellar media (ISM) and meteorites has unveiled a rich array of complex organic molecules, encompassing amino acids,^1^ sugars,^2^ purine bases,^3,4^ and short peptides.^5^ The identification of over 300 distinct compounds has spurred investigations into the origins of life and the fundamental chemical processes involved.^6^ Under the challenging conditions of the interstellar medium (ISM), characterized by low temperatures and high dilution, the formation of highly reactive species becomes essential for prebiotic organic reactions.^7–10^ While the formation mechanisms of such molecules are essentially “in the dark”, they must involve activating thermodynamically stable small molecules, such as CO, CO_2_, H_2_O, CH_4_, HCN, formaldehyde, simple alcohols, and various carboxylic acids.^11–13^ While our understanding of organic chemistry thrives in controlled (wet) laboratory environments, it faces limitations when extending into the much less well-understood conditions of the ISM. One challenging aspect is that the energy for initiating chemical reactions often is concentrated in terms of time and space, and is primarily provided by stellar energy beams.^14^ Hence, high-energy isomers of thermodynamically very stable molecules play a crucial role in the formation of biologically relevant compounds.^8,9,15^ The remarkable discovery of glycolaldehyde only in the year 2000 represents such a molecule.^16^ Its proposed formation mechanism, suggested only in 2018,^17^ involves the higher energy tautomer of formaldehyde, hydroxymethylene (H–C̈–OH), engaging in a highly facile, low-barrier process (Δ*H*^≠^ ≈ 1 kcal mol^−1^) with H_2_CO, even at the extremely low average temperature of space (∼2.7 K).^18^

Enols are considered crucial intermediates in the organic chemistry in space^19–22^ and several enol tautomers have been identified in the ISM. For instance, the simplest enol, vinyl alcohol (CH_2_=CHOH), was discovered in the analysis of microwave emissions from Sagittarius B2.35 in 2001.^23^ The gas phase stability of enols has also been validated through laboratory experiments, including those involving the enols of acetamide,^24^ acetic acid,^25^ glycolic acid,^26^ glycolaldehyde,^27^ and propionic acid.^28^ In solution, enols display high reactivity and isomerize rapidly into the more thermodynamically more stable keto tautomers through bimolecular acid–base reactions.^29^ In the gas phase, however, enols are considerably more stable^30^ due to significant energy barriers (ranging from 40 to 45 kcal mol^−1^)^30,31^ for [1,3]H-shifts. Consequently, enols can endure as long-lasting entities in the gas phase or in cold matrices, both of which model some aspects of the ISM (Scheme 1).^30^

**Scheme 1:**
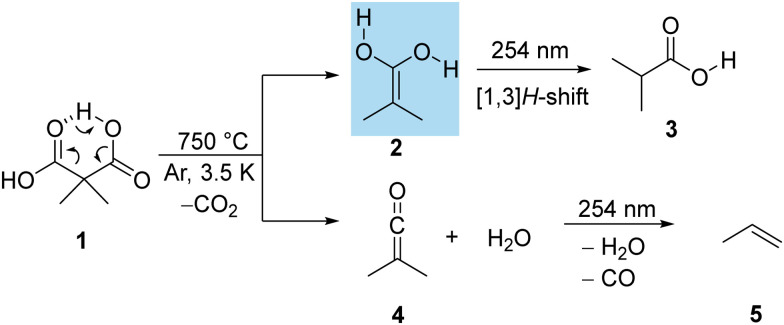
2-Methyl-prop-1-ene-1,1-diol (2) generated from dimethylmalonic acid (1) through pyrolysis and trapping in an argon matrix. Subsequent photorearrangement to isobutyric acid (3) and dimethylketene (4) as well as the photorearrangement of 4 to propene (5).

While the existence of dimethylketene 4 in ISM has not been proven to date, the presence of related molecules including parent ketene^32^ and methylketene,^33^ and their role as potential intermediates in the generation of prebiotic molecules has been suggested.^25,28^ That is, demonstrating the feasibility of 2 and 4 under conditions akin to the ISM (*T* ∼ 3 K and high dilution) would encourage their identification *via* radioastronomy; our methods of preparation can readily be transferred to complementary experimental setups including microwave emission experiments. Following the approach adopted in prior studies,^25^ we report here the generation of 2 alongside 4*via* pyrolysis of dimethylmalonic acid (1) as the precursor. The resulting molecules are subsequently captured in an argon matrix at a temperature of 3.5 K and characterized by IR and UV/vis spectroscopy and quantum chemical computations.

Enediol 2 was synthesized through the thermal decarboxylation of 1. This thermochemical process involved evaporating 1 at a temperature of 80 °C and subjecting it to pyrolysis in a quartz tube. We determined that the ideal pyrolysis temperature for enol generation is 750 °C; concurrently a significant amount of 4 forms as well. The pyrolysis products were subsequently condensed onto a cold matrix window at a temperature of 3.5 K, utilizing an abundant excess of argon as the condensing medium. Under these conditions, several characteristic bands were easily discernible in the spectroscopic analysis. Notably, the asymmetric CO_2_ stretching vibration at 2343 cm^−1^, the vibrational signatures corresponding to water and compound 3, and additional infrared bands attributed to 2 were readily identifiable (Fig. 1). The identification of 3 was verified through a comparison with a matrix-isolated IR spectrum of an authenticated sample. When subjected to irradiation at 254 nm, matrix-isolated 2 underwent [1,3]H-shift leading to 3. This transformation resulted in the disappearance of all IR absorption signals associated with 2. Simultaneously, new IR bands emerged, corresponding to isobutyric acid (3) (Fig. S2, ESI†).

**Fig. 1:**
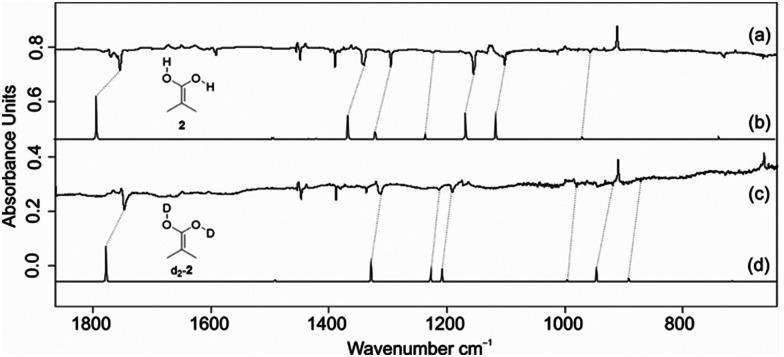
IR spectra after the pyrolysis of 1, followed by subsequent isolation in an argon matrix at 3.5 K. (a) Infrared difference spectra after irradiating 2 subsequent with a wavelength of 254 nm in an argon matrix at 3.5 K. Downward bands are attributed to 2 diminish, while bands assigned to 3 emerge after 20 min irradiation. (b) Computed infrared spectrum of 2 at B3LYP/def2-TZVP (unscaled). (c) Infrared difference spectra highlight the photoreactivity of deuterated d_2_-2 following irradiation with a wavelength of 254 nm in an argon matrix at 3.5 K. Downward bands corresponding to d_2_-2 decrease, while upward bands associated with d_2_-3 appear after 20 min of irradiation. (d) Computed infrared spectrum of deuterated d_2_-2 at B3LYP/def2-TZVP (unscaled).

The experimental IR spectrum of 2 exhibits a remarkable concordance with the computed IR spectrum of 2 obtained with B3LYP/def2-TZVP computations. After repeating the experiment using deuterated d_2_-1, the bands observed in d_2_-2 are also in good agreement with the computed spectrum; the isotopic shifts agree well. Specifically, the stretching vibration of the C=C bond at 1753 cm^−1^ exhibited a red-shift of 5 cm^−1^ (calculated: 14 cm^−1^) in d_2_-2. Analogously, the stretching vibrations of the OH groups in d_2_-2, at 2986 cm^−1^ and 2949 cm^−1^, were red-shifted by 662 cm^−1^ and 681 cm^−1^, respectively (calculated: 684 cm^−1^ and 730 cm^−1^). The good agreement between experimentally measured and computed shifts provides compelling evidence for the successful synthesis of 2. In the pyrolysis IR spectrum, we also identified bands corresponding to 4. The characteristic IR band at 2129 cm^−1^ (computed: 2183 cm^−1^) corresponds to the CCO stretching vibration of 4 (Fig. S1, ESI†).

The UV/Vis absorption spectrum of 2 displays a broad transition at 190 nm, in agreement with the computed TD-DFT spectrum that gives *λ*_max_ = 192 nm. This absorption band originates from a HOMO–LUMO+3 excitation, which correlates with a π → π* transition. In congruence with the IR experiments, the 190 nm band diminishes upon exposure to irradiation at 254 nm (Fig. 2).

**Fig. 2:**
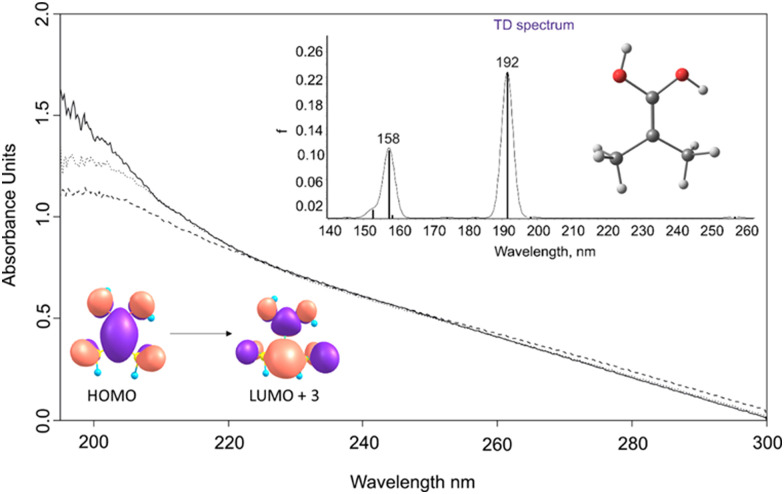
Solid line: UV/vis spectrum after pyrolysis of 1, followed by its isolation in an argon matrix at *T* = 3.5 K. Dotted line: The spectrum after irradiation with a wavelength of 254 nm for 6 min in an argon matrix at 3.5 K. Dashed line: The spectrum after irradiation with a wavelength of 254 nm for 20 min in an argon matrix at 3.5 K. Inset: Computed electronic transitions for 2 at TD-B3LYP/def2-TZVP.

As previously mentioned, pyrolysis led to the formation of 4 through the elimination of water from the enol in the gas phase. Unlike the enol tautomer of propionic acid,^28^ the generation of the enol from the precursor requires high activation energy (*E*_a_ = 31.1 kcal mol^−1^ computed at B3LYP/def2-TZVP), contributing to the formation of 4. Subsequent irradiation (*λ* = 254 nm) revealed the formation of propene (5). The distinct band at 1027 cm^−1^ can be assigned to the C=C wagging mode of 5; likewise, additional vibrations can be attributed to 5 (Fig. S4, ESI†). One question revolves around the mechanism of formation for 5, whether it occurs through a concerted mechanism from 4 or *via* a facile [1,2]H-shift from elusive dimethyl carbene (6), which may form through photochemical decarbonylation of 4. To date, only one free alkyl carbene has been characterized, namely 2-adamantyl carbene.^34^ In a previous examination of the rearrangement of 6 to propene, Evanseck and Houk theoretically estimated the activation energy for the [1,2]H-shift reaction to be 4.7 kcal mol^−1^.^35^ This low-energy process is probably also accompanied by some quantum mechanical tunneling (QMT) as suggested for the 1,2-hydrogen shift in methylchlorocarbene (in solution),^36^ a conclusion that would probably also hold for pure alkyl carbenes, thereby making their isolation even more challenging. As deuteration should slow QMT quite significantly, we also used d_6_-1 in an attempt to isolate d_6_-6, which, however, remained elusive, despite a somewhat higher computed barrier of 7.7 kcal mol^−1^.

To gain deeper insight into the thermochemistry of 2, we performed computations on the potential energy profile around 2 at DLPNO-CCSD(T)/cc-pVQZ//B3LYP/def2-TZVP+ZPVE at 0 K (Δ*H*_0_). Our computations reveal that 2 can exist in three distinct conformations, characterized by the orientations of the OH groups relative to the opposing C–O bond. These conformations are referred to as: s-*cis*, s-*trans*2ct, s-*trans*, s-*trans*2tt and s-*cis*, s-*cis*2cc. Among these conformers, 2ct is the most stable, with 2cc and 2tt being higher in energy by 0.7 and 2.0 kcal mol^−1^ (Fig. 3), respectively. The conformational isomerization from 2ct to 2tt involves an activation energy (TS1) of 3.6 kcal mol^−1^, while the activation energy for the 2ct to 2cc rotamerization is 4.5 kcal mol^−1^ (TS2). In our FVP experiments, 2ct is the predominant conformer in the matrix. Even with d_2_-1, no other conformer of 2 besides 2ct was observed. This is likely due to rapid (on the time scale of our experiments) QMT rotamerization, which is typical for OH groups.^25–27^ From the 2tt conformer, propionic acid conformer 3t can be generated through a [1,3]H-shift, which is associated with an activation barrier of 50.8 kcal mol^−1^ (TS3). The tautomerization from 2ct to 3c (the most thermodynamically stable conformer) involves an activation barrier of 51.3 kcal mol^−1^ (TS4). Apart from keto–enol tautomerization to 3c, 2ct can also dehydrate into 4 (4 + H_2_O) through transition state TS5, with an energy barrier of 47.2 kcal mol^−1^. Besides rapid rotation around the C–O bond, 2 exhibits limited reactivity due to the high and broad energy barriers surrounding it on its potential energy profile.^37^ This is demonstrated by the unchanged infrared bands of 2ct and d_2_-2ct when stored in the dark at 3.5 K for five days. As expected, under our pyrolysis reaction conditions, 4 readily formed. Fig. S1 (ESI†) reveals the emergence of 5 most likely originating from a concerted mechanism involving both a [1,2]H-shift and CO release from the ketene through TS6, featuring an activation barrier of 91.2 kcal mol^−1^. Given that this formation exclusively depends on irradiation and is associated with an excessively high activation barrier, it is apparent that 5 is exclusively accessible photochemically. It is also possible for the ketene to undergo a conversion to singlet carbene ^1^6, releasing CO upon photoexcitation. Carbene ^1^6 reacts to form propene through a [1,2]H-shift (TS7), facilitated by its low activation barrier 7.7 kcal mol^−1^ (which is also prone to QMT, *vide supra*). We can therefore not differentiate between the two photochemical paths and 6 remains elusive.

**Fig. 3:**
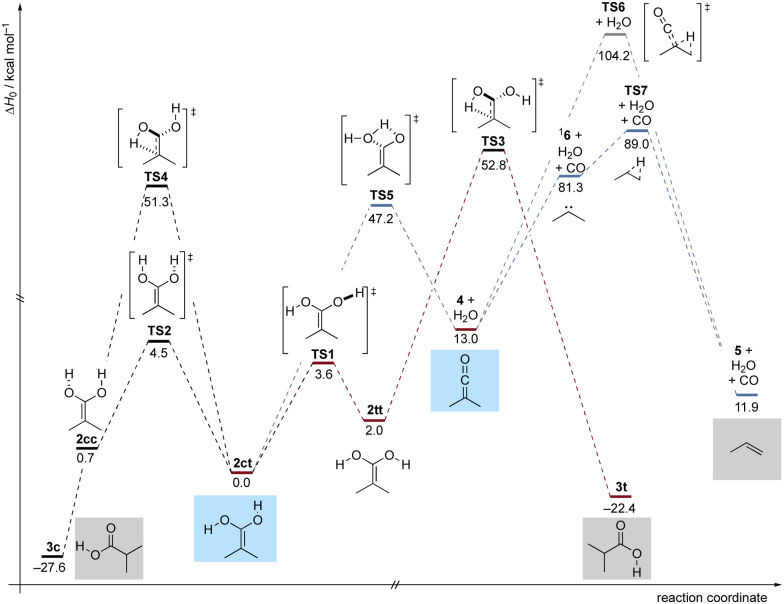
Potential energy profile (Δ*H*_0_) in kcal mol^−1^ of the reactions of dienol 2 at DLPNO-CCSD(T)/cc-pVQZ//B3LYP/def2-TZVP+ZPVE at 0 K.

Our results underscore the facile synthesis of acid tautomers in the gas phase, in particular, in the first preparation of 2 and its characterization by IR and UV/Vis spectroscopies as well as quantum chemical computations. Enol 2 therefore also constitutes a detectable interstellar gas-phase molecule. In the ISM, 2 may be accessible through the exothermic addition (by 13.0 kcal mol^−1^, Fig. 3) of water (*e.g.*, on ice grains) to 4. While 4 has not been identified in the ISM yet, the presence of parent ketene^32^ and very closely related methyl ketene^33^ has been confirmed. Finally, we also reveal the formation of propene, most likely through a photochemical mechanism from 4.

This project has received funding from the European Research Council (ERC) under the European Union's Horizon 2020 research and innovation programme (Advanced Grant No. 101054751 “COLDOC” to PRS). Views and opinions expressed are those of the authors only and do not necessarily reflect those of the European Union or the European Research Council. Neither the European Union nor the granting authority can be held responsible for them.

## Conflicts of interest

There are no conflicts to declare.

## Supplementary Material

CC-060-D4CC01140F-s001

## References

[cit1] Kvenvolden K., Lawless J., Pering K., Peterson E., Flores J., Ponnamperuma C., Kaplan I. R., Moore C. (1970). Evidence for Extraterrestrial Amino-acids and Hydrocarbons in the Murchison Meteorite. Nature.

[cit2] Furukawa Y., Chikaraishi Y., Ohkouchi N., Ogawa N. O., Glavin D. P., Dworkin J. P., Abe C., Nakamura T. (2019). Extraterrestrial ribose and other sugars in primitive meteorites. Proc. Natl. Acad. Sci. U. S. A..

[cit3] Callahan M. P., Smith K. E., Cleaves H. J., Ruzicka J., Stern J. C., Glavin D. P., House C. H., Dworkin J. P. (2011). Carbonaceous meteorites contain a wide range of extraterrestrial nucleobases. Proc. Natl. Acad. Sci. U. S. A..

[cit4] Stoks P. G., Schwartz A. W. (1979). Uracil in carbonaceous meteorites. Nature.

[cit5] Shimoyama A., Ogasawara R. (2002). Dipeptides and Diketopiperazines in the Yamato-791198 and Murchison Carbonaceous Chondrites. Origins Life Evol. Biospheres.

[cit6] https://cdms.astro.uni-koeln.de/classic/molecules. (accessed March 05, 2024)

[cit7] Miller S. L., Urey H. C. (1959). Organic Compound Synthesis on the Primitive Earth. Science.

[cit8] Bada J. L. (2013). New insights into prebiotic chemistry from Stanley Miller's spark discharge experiments. Chem. Soc. Rev..

[cit9] Muñoz Caro G. M., Meierhenrich U. J., Schutte W. A., Barbier B., Arcones Segovia A., Rosenbauer H., Thiemann W. H. P., Brack A., Greenberg J. M. (2002). Amino acids from ultraviolet irradiation of interstellar ice analogues. Nature.

[cit10] Ruiz-Mirazo K., Briones C., de la Escosura A. (2014). Prebiotic systems chemistry: new perspectives for the origins of life. Chem. Rev..

[cit11] Arumainayagam C. R., Garrod R. T., Boyer M. C., Hay A. K., Bao S. T., Campbell J. S., Wang J., Nowak C. M., Arumainayagam M. R., Hodge P. J. (2019). Extraterrestrial prebiotic molecules: photochemistry *vs.* radiation chemistry of interstellar ices. Chem. Soc. Rev..

[cit12] Irvine W. M. (1998). Extraterrestrial Organic Matter: A review. Origins Life Evol. Biospheres.

[cit13] Hollis J. M., Lovas F. J., Remijan A. J., Jewell P. R., Ilyushin V. V., Kleiner I. (2006). Detection of Acetamide (CH_3_CONH_2_): The Largest Interstellar Molecule with a Peptide Bond. Astrophys. J..

[cit14] Arumainayagam C. R., Garrod R. T., Boyer M. C., Hay A. K., Bao S. T., Campbell J. S., Wang J., Nowak C. M., Arumainayagam M. R., Hodge P. J. (2019). Extraterrestrial prebiotic molecules: photochemistry vs. radiation chemistry of interstellar ices. Chem. Soc. Rev..

[cit15] Miller S. L., Urey H. C. (1959). Organic compound synthesis on the primitive Earth: Several questions about the origin of life have been answered, but much remains to be studied. Science.

[cit16] Hollis J. M., Lovas F. J., Jewell P. R. (2000). Interstellar Glycolaldehyde: The First Sugar. Astrophys. J..

[cit17] Eckhardt A. K., Linden M. M., Wende R. C., Bernhardt B., Schreiner P. R. (2018). Gas-phase sugar formation using hydroxymethylene as the reactive formaldehyde isomer. Nat.
Chem..

[cit18] Schreiner P. R., Reisenauer H. P., Pickard F. C. T., Simmonett A. C., Allen W. D., Mátyus E., Császár A. G. (2008). Capture of hydroxymethylene and its fast disappearance through tunnelling. Nature.

[cit19] Ménez B., Pisapia C., Andreani M., Jamme F., Vanbellingen Q. P., Brunelle A., Richard L., Dumas P., Réfrégiers M. (2018). Abiotic synthesis of amino acids in the recesses of the oceanic lithosphere. Nature.

[cit20] Chyba C. F., Thomas P. J., Brookshaw L., Sagan C. (1990). Cometary delivery of organic molecules to the early Earth. Science.

[cit21] Ehrenfreund P., Charnley S. B. (2000). Organic Molecules in the Interstellar Medium, Comets, and Meteorites: A Voyage from Dark Clouds to the Early Earth. Annu. Rev. Astron. Astrophys..

[cit22] Pearson V. K., Sephton M. A., Kearsley A. T., Bland P. A., Franchi I. A., Gilmour I. (2002). Clay mineral-organic matter relationships in the early solar system. Meteorit. Planet. Sci..

[cit23] Turner B. E., Apponi A. J. (2001). Microwave detection of interstellar vinyl alcohol, CH_2_=CHOH. Astrophys. J..

[cit24] Mardyukov A., Keul F., Schreiner P. R. (2020). Preparation and characterization of the enol of acetamide: 1-aminoethenol, a high-energy prebiotic molecule. Chem. Sci..

[cit25] Mardyukov A., Eckhardt A. K., Schreiner P. R. (2020). 1,1-Ethenediol: The Long Elusive Enol of Acetic Acid. Angew. Chem., Int. Ed..

[cit26] Mardyukov A., Keul F., Schreiner P. R. (2021). 1,1,2-Ethenetriol: The Enol of Glycolic Acid, a High-Energy Prebiotic Molecule. Angew. Chem., Int. Ed..

[cit27] Mardyukov A., Wende R. C., Schreiner P. R. (2023). Matrix isolation and photorearrangement of *cis*- and *trans*-1,2-ethenediol to glycolaldehyde. Chem. Commun..

[cit28] Danho A., Mardyukov A., Schreiner P. R. (2023). The enol of propionic acid. Chem. Commun..

[cit29] Meyer K. H. (1914). Über das Gleichgewicht desmotroper Verbindungen in verschiedenen Lösungsmitteln (Über Keto-Enol-Tautomerie. IX). Ber. Dtsch. Chem. Ges..

[cit30] Taatjes C. A., Hansen N., McIlroy A., Miller J. A., Senosiain J. P., Klippenstein S. J., Qi F., Sheng L., Zhang Y., Cool T. A. (2005). Enols are common intermediates in hydrocarbon oxidation. Science.

[cit31] Kleimeier N. F., Kaiser R. I. (2021). Interstellar Enolization-Acetaldehyde (CH_3_CHO) and Vinyl Alcohol (H_2_CCH(OH)) as a Case Study. ChemPhysChem.

[cit32] Turner B. (1977). Microwave detection of interstellar ketene. Astrophys. J..

[cit33] Bermúdez C., Tercero B., Motiyenko R., Margulès L., Cernicharo J., Ellinger Y., Guillemin J.-C. (2018). The millimeter-wave spectrum of methyl ketene and the astronomical search for it. Astron. Astrophys..

[cit34] Bally T., Matzinger S., Truttmann L., Platz M. S., Morgan S. (1994). Matrix Spectroscopy of 2-Adamantylidene, a Dialkylcarbene with Singlet Ground State. Angew. Chem., Int. Ed. Engl..

[cit35] Raghavachari K., Whiteside R. A., Pople J. A., Schleyer P. V. R. (1981). Molecular orbital theory of the electronic structure of organic molecules. 40. Structures and energies of *C*_1_–*C*_3_ carbocations including effects of electron correlation. J. Am. Chem. Soc..

[cit36] Dix E. J., Herman M. S., Goodman J. L. (1993). The 1,2-hydrogen rearrangement of methylchlorocarbene: contribution of quantum mechanical tunneling. J. Am. Chem. Soc..

[cit37] Ley D., Gerbig D., Schreiner P. R. (2012). Tunnelling control of chemical reactions – the organic chemist's perspective. Org. Biomol. Chem..

