# Supplementary material for: The enol of isobutyric acid
Source: Chem Commun (Camb). 2024 Apr 16;60(39):5161–4. doi: 10.1039/d4cc01140f (PMC11080965; doi:10.1039/d4cc01140f)
Supplement: CC-060-D4CC01140F-s001 [file CC-060-D4CC01140F-s001.pdf]

Electronic Supplementary Material (ESI) for ChemComm.
This journal is © The Royal Society of Chemistry 2024

The Enol of Isobutyric Acid

Akkad Danho, Artur Mardyukov, and Peter R. Schreiner*

Table of Contents

Experimental Procedures 2

Synthesis 3

Figure S1: 6

Figure S2: 7

Figure S3: 7

Figure S4: 8

Table S1: 9

Table S2: 10

Table S3: 11

References 12

Optimized structures 13

Experimental Procedures

Matrix Apparatus Design. A Sumitomo cryostat system consisting of an RDK 408D2 closed-cycle refrigerator cold head and an F-70 compressor unit was used for matrix isolation experiments. A polished CsI window was mounted in the cold head sample holder. The sample holder, connected with silicon diodes for temperature measurements, was covered by a vacuum shroud, which was equipped with KBr windows to allow for IR measurements. In some experiments BaF_2 windows were used due to their higher transparency when measuring UV/vis spectra. The sample and the host gas (Ar, purity of 99.999%) were co-deposited at 3.5 K. All spectral data were collected at 3.5 K. The pyrolysis zone was equipped with a heatable 90 mm long quartz tube (inner diameter 7 mm), controlled by a Ni/CrNi thermocouple. The travel distance of the sample from the pyrolysis zone to the matrix was ~45 mm. Ar was stored in a 2 L gas balloon, which was evacuated and filled three times before every experiment. The sample was evaporated from a Schlenk tube at 80 °C (water) and reduced pressure (~3×10^{-6} mbar) and co-deposited with a high excess of argon on both sides of the matrix window in the dark (preventing unwanted photochemistry) at a rate of ~1 mbar min^{-1}, based on the pressure inside the Ar balloon. Pyrolyses were carried out at 750 °C. IR spectra were recorded between 7000 and 350 cm^{-1} with a resolution of 0.7 cm^{-1} with a Bruker Vertex 70 FTIR spectrometer. A spectrum of the cold matrix window before deposition was used as background spectrum for the subsequent IR measurements. UV/vis spectra were recorded between 190 and 800 nm with a resolution of 1 nm with a Jasco V-760 spectrophotometer. A high-pressure-mercury lamp equipped with a monochromator (LOT Quantum Design) or a low-pressure-mercury lamp (Gräntzel) fitted with a Vycor filter were used for irradiation of the matrix during photochemical experiments.

Computations. All DFT computations were performed with the Gaussian 16,[1] Revision C.01 (full citations for electronic structure codes are given at the end of this document) at the B3LYP/def2-TZVP[2-3] level of theory. The keywords Opt and Freq=NoRaman were used for the characterization of minima on the PES. For transition structures the keyword Opt=(ts,tight,calcfc,noeigen) was used. UV/Vis absorptions were computed by using the keyword td(50-50,nstates=10).

Synthesis

Diethyl dimethylmalonate-d_6

To a solution of 0.550 g (3.43 mmol, 1.00 equiv) diethyl malonate in 7 mL anhydrous *N*,*N*-dimethylformamide 1.42 g (10.27 mmol, 3.33 equiv) K_2CO_3 was added. The reaction was stirred at room temperature for 1 h and 0.440 mL (1.000, 7.03 mmol, 2.05 equiv) iodmethane-d_3 was added and the reaction mixture was stirred at room temperature for 4 d. The remaining salts were filtered and the yellow solution was quenched with 6 mL (5% wt) $Na_2S_2O_3$-solution and extracted with ethyl acetate. The combined organic layers were dried with $MgSO_4$, filtered, and the solvent was removed under reduced pressure, giving 0.454 g (2.32 mmol) of diethyl dimethylmalonate-d_6 in 68% yield. The residue contained DMF, yet proved inconsequential for the subsequent step, allowing for the utilization of the crude product without additional purification.

^{1}H-NMR (400 MHz, $CDCl_3$): δ/ppm = 4.18 (q, *J* = 8.0 Hz, 4H, 2 CH_2), 1.25 (t, *J* = 8 Hz, 6H, 2 CH_3).

^{13}C-NMR (100 MHz, $CDCl_3$): δ/ppm = 173.1, 170.3, 61.5, 61.3, 46.1, 14.2, 14.2.

HRMS: m/z = 217.1316 $[M+Na]^+$, 217.1317 (calc).

Dimethylmalonic acid-d_6

0.302 g (1.57 mmol, 1.00 equiv) of crude diethyl dimethylmalonate-d_6 and 0.264 g (4.71 mmol, 3.00 equiv) KOH were dissolved in 2 mL H_2O and the reaction mixture was refluxed for 3 h. After cooling to room temperature the reaction mixture was acidified with conz. HCl-solution to a pH of 2 and extracted with acetone. The combined organic layers were dried with $MgSO_4$, filtered, and the solvent was removed under reduced pressure, giving 0.180 g (1.30 mmol) of dimethylmalonic acid-d_6 in 84 % yield.

^{1}H-NMR (400 MHz, CD_3OD): δ/ppm = /.

^{13}C-NMR (100 MHz, CD_3OD): δ/ppm = 177.0, 50.3, 22.6.

HRMS: m/z = 161.0695 $[M+Na]^+$, 161.0691 (calc).

Diethyl dimethylmalonate-d_6

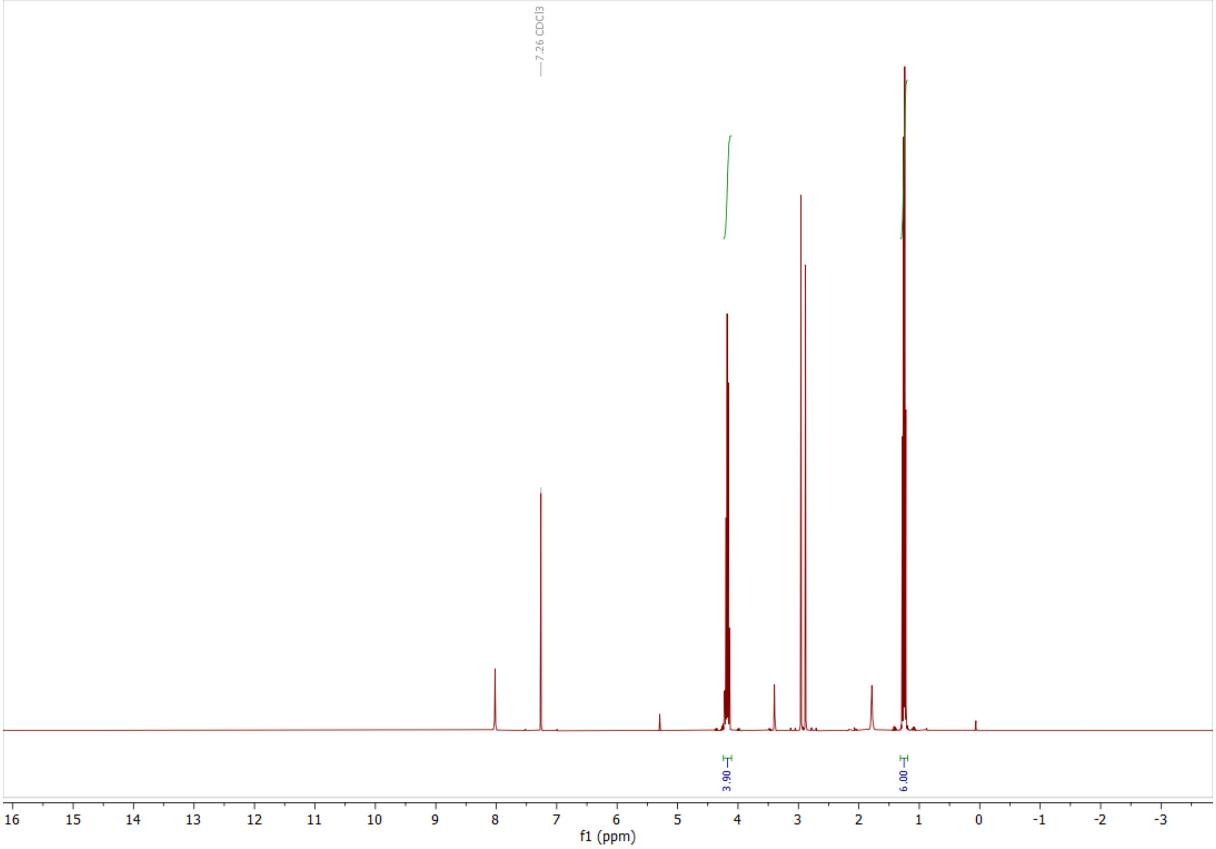

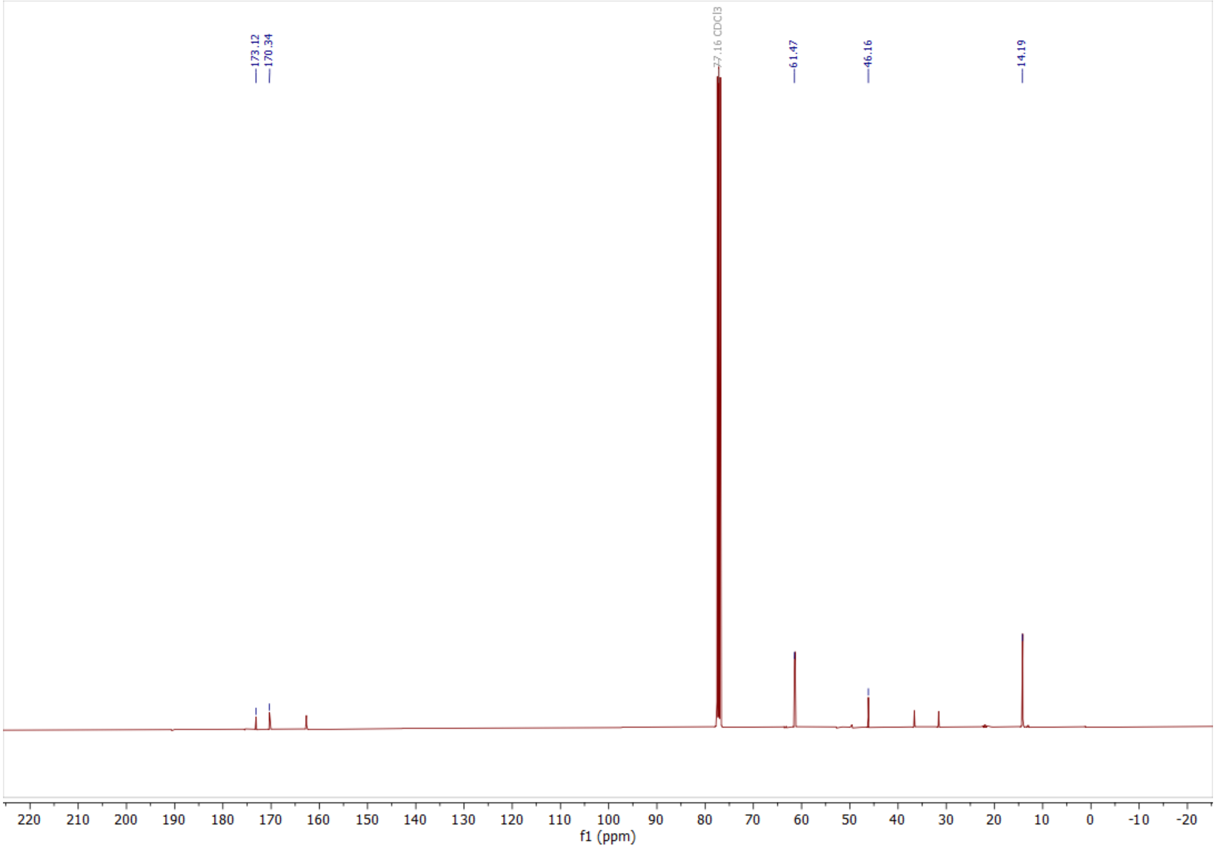

Dimethylmalonic acid-d_6

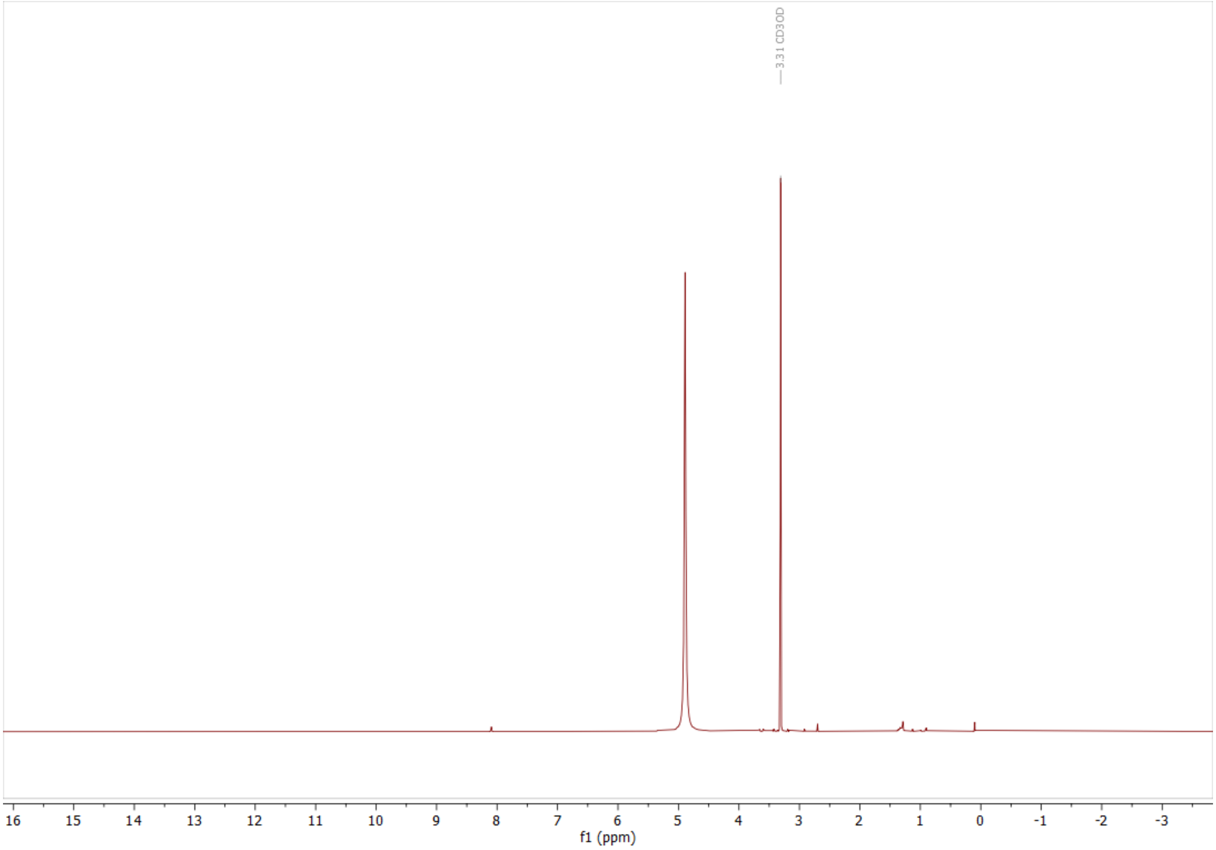

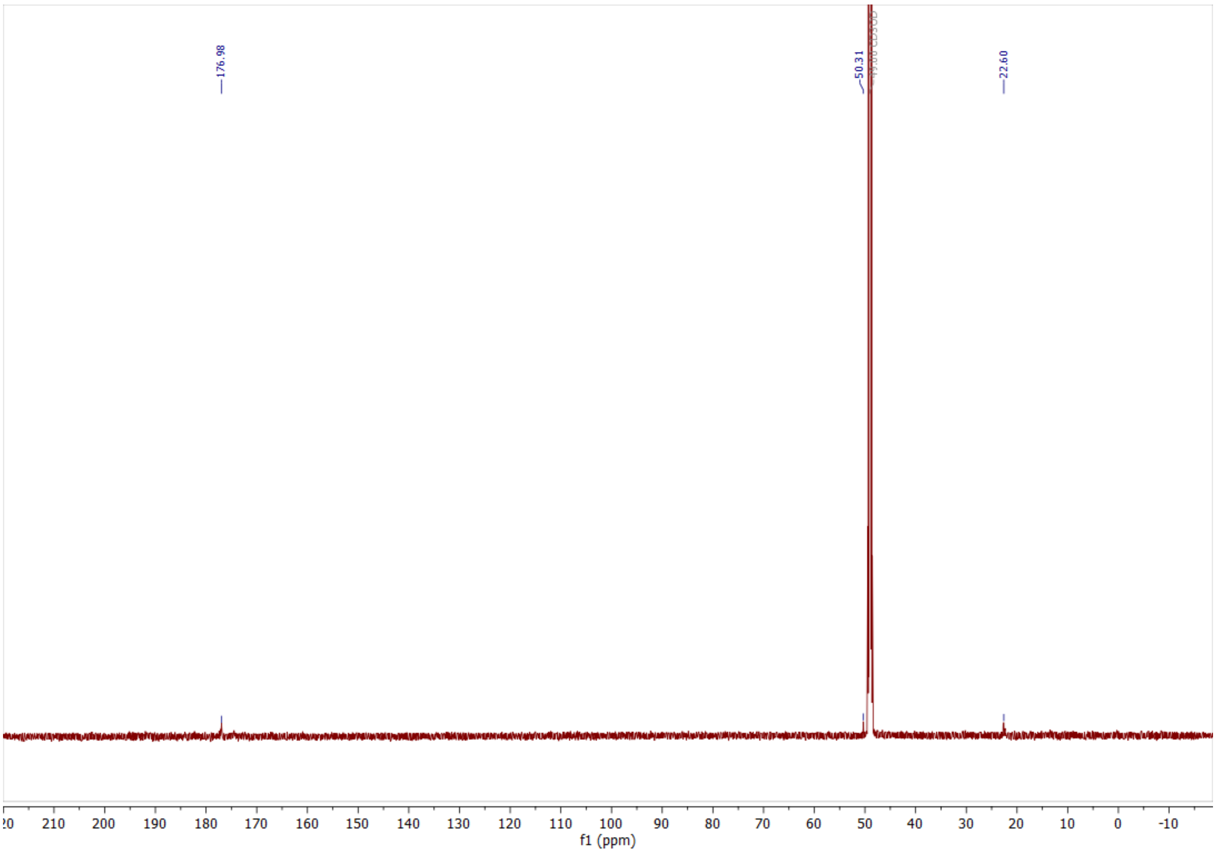

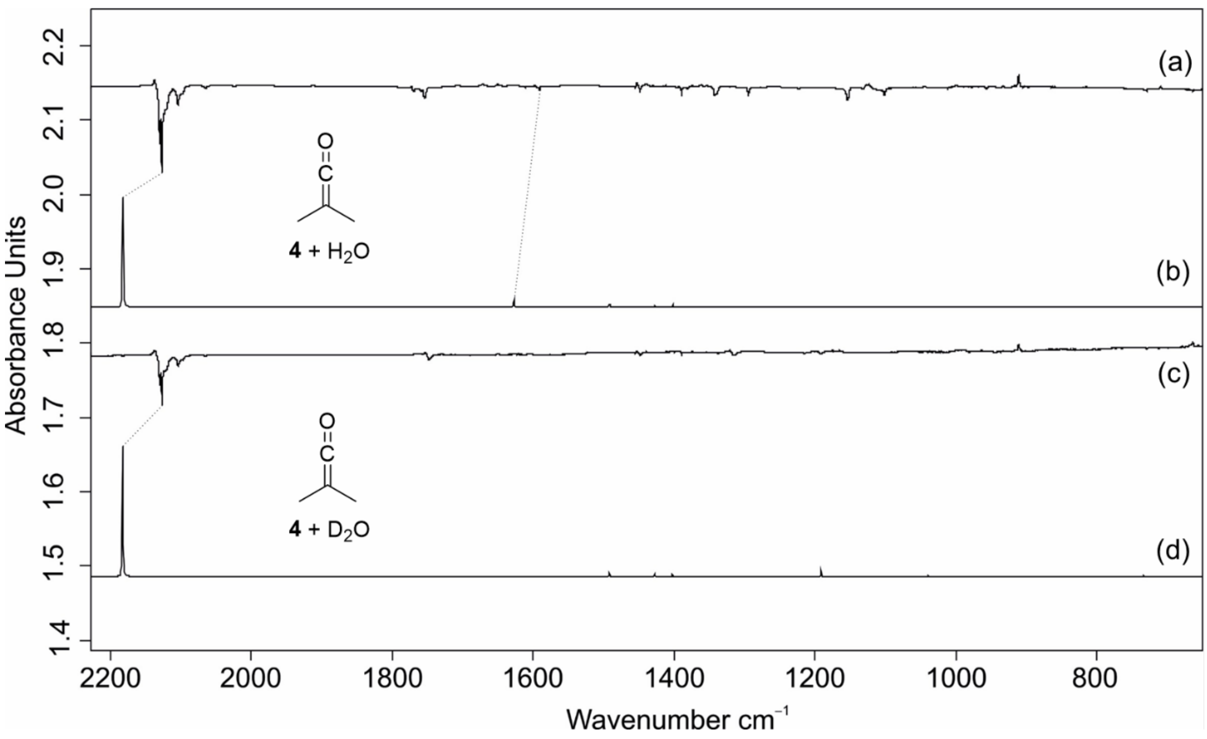

Figure S1: IR spectra showing the pyrolysis product of **1** with subsequent trapping in an argon matrix at 3.5 K (a) IR difference spectra showing the photochemistry of **4** after irradiation with λ = 254 nm in argon at 3.5 K. Downward bands assigned to **4** disappear after 20 min irradiation time. (b) IR spectrum of **4** computed at B3LYP/def2-TZVP. (c) IR difference spectra showing the photochemistry of d_1-**4** after irradiation with λ = 254 nm in argon at 3.5 K. Downward bands assigned to d_1-**4** disappear after 20 min irradiation time. (d) IR spectrum of d_1-**4** computed at B3LYP/def2-TZVP.

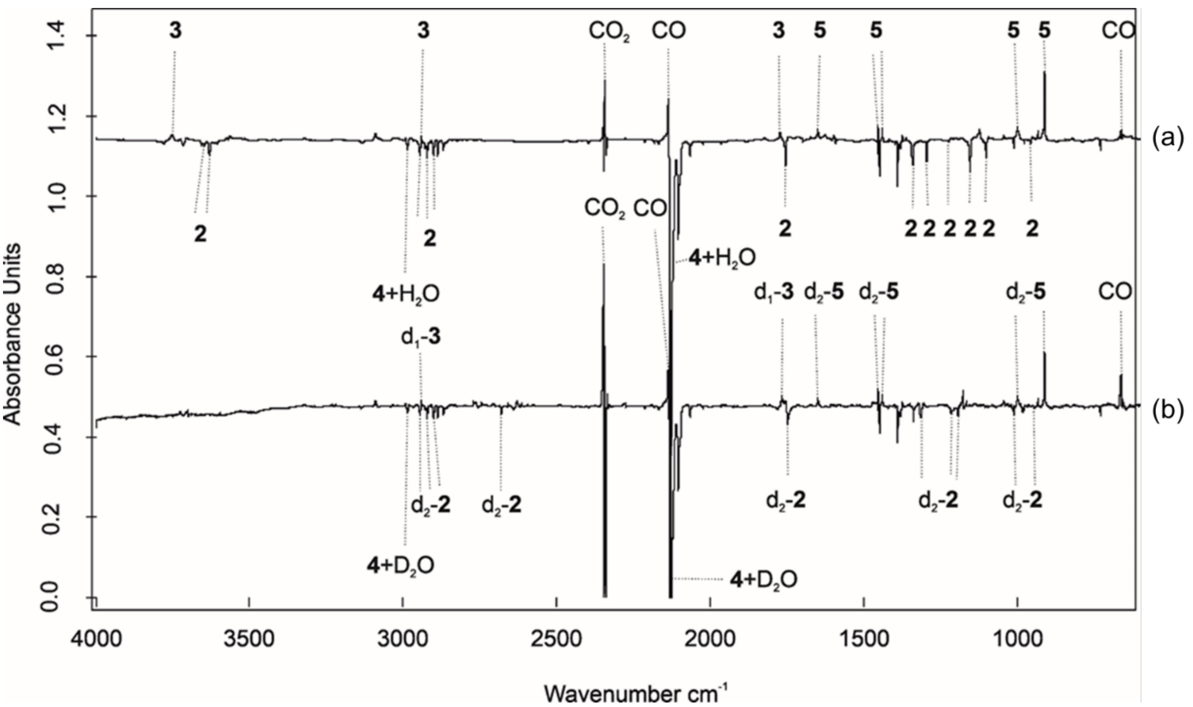

Figure S2: (a) IR difference spectra showing the photochemistry of **1** after irradiation with λ = 254 nm in argon at 3.5 K. (b) IR difference spectra showing the photochemistry of d$_2$-**1** after irradiation with λ = 254 nm in argon at 3.5 K.

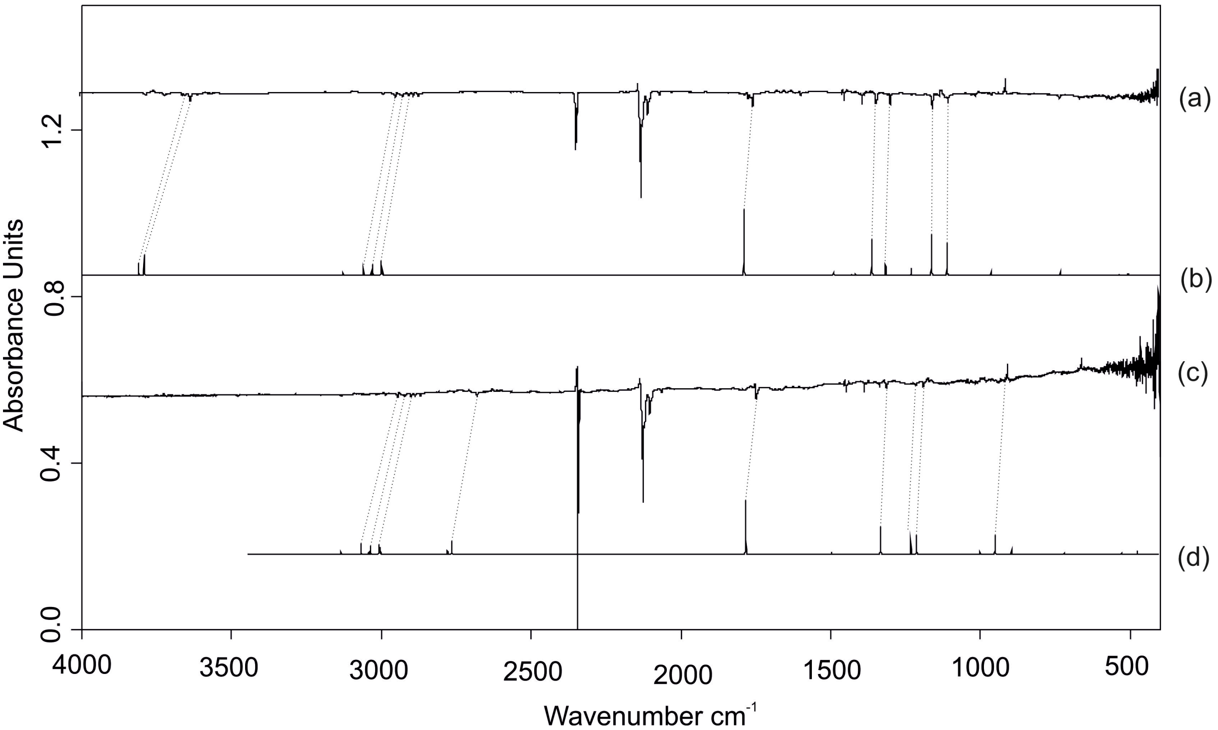

Figure S3: IR spectra showing the pyrolysis product of **1** with subsequent trapping in an argon matrix at 3.5 K. (a) IR difference spectra showing the photochemistry of **2** after irradiation with λ = 254 nm in argon at 3.5 K. Downward bands assigned to **2** disappear while upward bands assigned **3** appear after 20 min irradiation time. (b) IR spectrum of **2** computed at B3LYP/def2-TZVP. (c) IR difference spectra showing the photochemistry of d$_2$-**2** after irradiation with λ = 254 nm in argon at 3.5 K. Downward bands assigned to d$_2$-**2** disappear

while upward bands assigned to d_2-**3** appear after 20 min irradiation time. (d) IR spectrum of d_2-**2** computed at B3LYP/def2-TZVP.

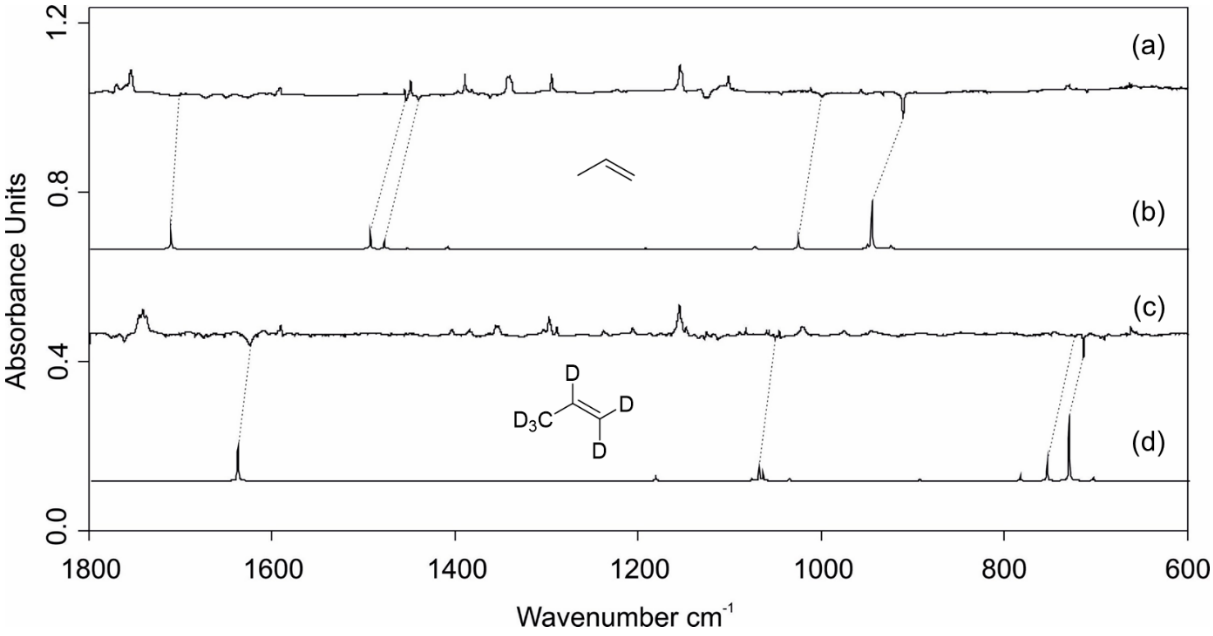

Figure S4: IR spectra showing the pyrolysis product of **1** with subsequent trapping in an argon matrix at 3.5 K. (a) IR difference spectra showing the photochemistry of **2** after irradiation with λ = 254 nm in argon at 3.5 K. Downward bands assigned to propene appear after 20 min irradiation time. (b) IR spectrum of **2** computed at B3LYP/def2-TZVP. (c) IR difference spectra showing the photochemistry of d_6-**2** after irradiation with λ = 254 nm in argon at 3.5 K. Downward bands assigned to d_6-propylene appear after 20 min irradiation time. (d) IR spectrum of d_2-**2** computed at B3LYP/def2-TZVP.

Table S1: Experimental (Ar matrix, 3.5 K) and computed IR frequencies of **2** and d_2-**2** band origins in cm^{-1}, computed intensities (km mol^{-1}) in parantheses.

Mode	**2** Computed[a]	**2** Ar 3.5 K[b]	d_2-**2** Computed[a]	d_2-**2** Ar 3.5 K[b]	Assignment
1	3812 (46.9)	3648 (w)	3128 (10.5)	2986 (w)	O–H stretching
2	3792 (94.9)	3630 (m)	3062 (50.7)	2949 (m)	O–H stretching
3	3128 (10.4)	2989 (w)	3036 (17.7)	2924 (m)	C–H (CH_3) stretching
4	3062 (49.5)	2946 (m)	3030 (45.2)	2903 (m)	C–H (CH_3) stretching
5	3036 (17.7)	2918 (m)	3002 (67.4)	2889 (m)	C–H (CH_3) stretching
6	3030 (45.2)	2898 (m)	2998 (41.7)	2871 (m)	C–H (CH_3) stretching
7	3002 (68.1)	2888 (m)	2773 (27.2)	-	C–H (CH_3) stretching
8	2998 (41.0)	2868 (m)	2760 (61.7)	2680 (m)	C–H (CH_3) asym.–stretching
9	1792 (254.3)	1755 (s)	1778 (250.8)	1750 (s)	C=C stretching
10	1509 (0.5)	-	1506 (2.1)	-	C–H (CH_3) bending
11	1494 (10.0)	1453 (m)	1493 (6.9)	-	C–H (CH_3) bending
12	1492 (13.2)	1449 (m)	1492 (13.0)	1448 (m)	C–H (CH_3) twisting
13	1471 (2.5)	-	1471 (2.3)	-	C–H (CH_3) twisting
14	1432 (4.8)	-	1431 (1.4)	-	C–C stretching
15	1420 (9.2)	1387 (m)	1416 (1.3)	-	C–C asym.–stretching
16	1366 (151.0)	1337 (s)	1329 (132.3)	1315 (s)	C–O stretching
17	1319 (67.5)	1294 (s)	1228 (69.6)	1212 (m)	O–H wagging
18	1234 (28.9)	1227 (w)	1209 (106.4)	1191 (s)	O–H and C–C in pl.
19	1166 (177.8)	1156 (s)	1099 (0.3)	-	O–H wagging
20	1115 (126.6)	1101 (s)	1011 (0.7)	-	C–CH_3 twisting
21	1099 (0.5)	-	997 (19.8)	983 (w)	C–H (CH_3) twisting
22	1011 (0.2)	-	985 (2.8)	-	C–H (CH_3) asym.–twisting
23	986 (2.3)	-	947 (105.5)	943 (w)	C–CH_3 asym.–twisting
24	968 (22.8)	958 (w)	892 (35.6)	-	C–CH_3 asym.–twisting
25	737 (15.7)	734 (w)	717 (10.7)	-	C–C and C–O stretching
26	648 (3.2)	-	645 (4.9)	-	OCO in pl. def.
27	540 (5.5)	-	526 (6.6)	-	C=C in pl. def.
28	511 (14.3)	-	473 (15.7)	-	C–O wagging
29	359 (1.6)	-	355 (0.9)	-	C–CH_3 wagging
30	329 (31.4)	-	311 (4.5)	-	O–H wagging
31	304 (3.1)	-	267 (5.0)	-	O–H wagging
32	273 (9.4)	-	253 (21.7)	-	C=C in pl. def.
33	267 (118.5)	-	204 (35.2)	-	O–H wagging

34	158 (8.0)	-	155 (4.3)	-	C–CH_3 twisting
35	126 (22.2)	-	121 (15.3)	-	C–CH_3 twisting
36	93 (30.0)	-	87 (34.5)	-	C–CH_3 twisting

[a] B3LYP/def2-TZVP, harmonic approximation, unscaled frequencies, intensities (in parentheses) in km mol^{-1}. [b] Experiment: argon matrix, 3.5 K; approximate relative intensities (w: weak, m: medium, s: strong).

Table S2: Experimental (Ar matrix, 3.5 K) and computed IR frequencies of **4** + H_2O and **4** + D_2O band origins in cm^{-1}, computed intensities (km mol^{-1}) in parantheses.

Mode	**4** + H_2O Computed[a]	**4** Ar 3.5 K[b]	**4** + D_2O Computed[a]	**4** + D_2O Ar 3.5 K[b]	Assignment
1	3052 (48.4)	-	3052 (48.4)	-	C–H (CH_3) wagging
2	3012 (55.6)	-	3012 (55.6)	-	C–H (CH_3) stretching
3	2840 (45.4)	-	2840 (45.3)	-	H–O asym.stretching
4	2183 (760.7)	2129 (s)	2182 (760.7)	2129 (s)	C=C stretching
5	1192 (36.5)	-	1191 (36.5)	-	O–H wagging
6	240 (127.0)	-	240 (127.1)	-	O–H rocking

[a] B3LYP/def2-TZVP, harmonic approximation, unscaled frequencies, intensities (in parentheses) in km mol^{-1}. [b] Experiment: argon matrix, 3.5 K; approximate relative intensities (w: weak, m: medium, s: strong).

Table S3: Experimental (Ar matrix, 3.5 K) and computed IR frequencies of **propylene** and **d_6-propylene** band origins in cm^{-1}, computed intensities (km mol^{-1}) in parantheses.

Mode	**propylene** Computed[a]	**propylene** Ar, 3.5 K[b]	**D_6-propylene** Computed[a]	**D_6-propylene** Ar, 3.5 K[b]	Assignment
1	3213 (16.9)	-	2393 (8.1)	-	C–H (CH_2) stretching
2	3132 (2.7)	-	2320 (5.3)	-	C–H (CH) stretching
3	3126 (30.5)	-	2294 (10.4)	-	C–H (CH_3) stretching
4	3094 (10.3)	-	2278 (4.8)	-	C–H (CH_2) asym.stretching
5	3017 (24.4)	-	2169 (10.3)	-	C–H (CH_3) asym.stretching
6	1712 (15.2)	1709 (w)	1639 (16.5)	1625 (m)	C=C stretching
7	1494 (15.2)	1453 (m)	1183 (2.5)	1051 (w)	C–C bending
8	1479 (7.4)	1440 (w)	1078 (0.6)	-	C–H (CH_3) wagging
9	1453 (0.9)	-	1070 (7.3)	-	C–H (CH_3) bending
10	1410 (2.0)	-	1066 (3.8)	-	C–H (CH_3) twisting
11	1332 (0.0)	-	1037 (0.8)	-	C–C bending in pl.
12	1194 (0.3)	-	949 (0.0)	-	C–C bending in pl.
13	1074 (2.6)	-	895 (0.9)	-	C–C wagging
14	1027 (11.3)	1000 (w)	785 (2.0)	-	C–C wagging in pl.
15	951 (3.1)	-	756 (7.9)	-	C=C wagging
16	946 (48.7)	910 (s)	732 (28.1)	715 (s)	C=C in pl. def.
17	925 (3.2)	-	706 (1.5)	-	C–C in pl. def.
18	592 (12.9)	-	443 (7.6)	-	C–H in pl. def.
19	426 (1.0)	-	349 (0.7)	-	C–C rocking
20	206 (0.5)	-	152 (0.2)	-	C–CH_3 twisting

[a] B3LYP/def2-TZVP, harmonic approximation, unscaled frequencies, intensities (in parentheses) in km mol^{-1}. [b] Experiment: argon matrix, 3.5 K; approximate relative intensities (w: weak, m: medium, s: strong).

References

1. M. J. Frisch, G. W. T., H. B. Schlegel, G. E. Scuseria, M. A. Robb, J. R. Cheeseman, G. Scalmani,; V. Barone, G. A. P., H. Nakatsuji, X. Li, M. Caricato, A. V. Marenich, J. Bloino, B. G. Janesko,; R. Gomperts, B. M., H. P. Hratchian, J. V. Ortiz, A. F. Izmaylov, J. L. Sonnenberg, Williams, F.; Ding, F. L., F. Egidi, J. Goings, B. Peng, A. Petrone, T. Henderson, D. Ranasinghe, V. G.; Zakrzewski, J. G., N. Rega, G. Zheng, W. Liang, M. Hada, M. Ehara, K. Toyota, R. Fukuda, J.; Hasegawa, M. I., T. Nakajima, Y. Honda, O. Kitao, H. Nakai, T. Vreven, K. Throssell, J. A.; Montgomery Jr., J. E. P., F. Ogliaro, M. J. Bearpark, J. J. Heyd, E. N. Brothers, K. N. Kudin, V.; N. Staroverov, T. A. K., R. Kobayashi, J. Normand, K. Raghavachari, A. P. Rendell, J. C. Burant, S.; S. Iyengar, J. T., M. Cossi, J. M. Millam, M. Klene, C. Adamo, R. Cammi, J. W. Ochterski, R. L.; Martin, K. M., O. Farkas, J. B. Foresman, D. J. Fox,, Gaussian 16, revision C. 01. Gaussian, Inc., Wallingford CT: 2016.

2. Becke, A. D., Density-functional exchange-energy approximation with correct asymptotic behavior. *Phys. Rev. A.* **1988,** *38* (6), 3098.

3. Becke, A., Density-Functional Thermochemistry. III. The Role of Exact Exchange. J. Chem. Phys., 98: 5648-5652. 1993.

Optimized structures (distances in Å), electronic energies (in hartree) and zero-point vibrational energies (ZPVE, in hartree).

3c: Isobutyic acid *cis* (C_1)

O	1.685683000	0.693024000	-0.493444000
C	0.782312000	-0.191651000	-0.001543000
C	-0.635279000	0.177990000	-0.390541000
C	-1.560301000	-1.034017000	-0.315548000
C	-1.119963000	1.330275000	0.508048000
O	1.110922000	-1.128699000	0.678671000
H	2.557789000	0.412666000	-0.173703000
H	-0.592946000	0.548807000	-1.418263000
H	-2.571321000	-0.750589000	-0.612999000
H	-1.596607000	-1.432174000	0.698767000
H	-1.222493000	-1.835315000	-0.973754000
H	-2.126035000	1.631526000	0.212320000
H	-0.466526000	2.199285000	0.430238000
H	-1.155313000	1.015611000	1.553087000

E (DLPNO-CCSD(T)) = –307.230737
ZPVE (B3LYP) = 0.118390

3t: Isobutyic acid *trans* (C_1)

O	1.799384000	0.602644000	-0.420963000
C	0.802388000	-0.215636000	0.016378000
C	-0.612032000	0.194275000	-0.385712000
C	-1.554116000	-1.006741000	-0.368961000
C	-1.099151000	1.314419000	0.551660000
O	1.071656000	-1.161327000	0.699016000
H	1.442386000	1.303853000	-0.980495000
H	-0.568534000	0.595544000	-1.405466000
H	-2.560419000	-0.699558000	-0.659007000
H	-1.596649000	-1.444491000	0.628224000
H	-1.219910000	-1.783561000	-1.057265000
H	-2.098155000	1.639750000	0.257841000
H	-0.442099000	2.185935000	0.530931000
H	-1.147480000	0.954092000	1.580625000

E (DLPNO-CCSD(T)) = –307.2223619

ZPVE (B3LYP) = 0.118067

2ct: Enol *cis*, *anti* (C_1)

O	-1.508315000	1.093946000	0.011103000
C	-0.690825000	-0.004619000	-0.005080000
O	-1.444126000	-1.132906000	-0.052473000
C	0.644413000	-0.015046000	0.002057000
C	1.410767000	-1.307821000	0.031606000
H	-0.984555000	1.883894000	0.182898000
H	-2.356186000	-0.887803000	0.146113000
H	0.751541000	-2.169531000	0.110793000
H	2.103729000	-1.331804000	0.880058000
H	2.017413000	-1.429482000	-0.872876000
C	1.464943000	1.244610000	-0.027204000
H	2.027358000	1.382711000	0.903064000
H	2.201998000	1.205826000	-0.836105000
H	0.882435000	2.155128000	-0.191256000

E (DLPNO-CCSD(T)) = –307.186736

ZPVE (B3LYP) = 0.116370

2cc: Enol *cis*, *cis* (C_2)

O	1.145797034	-0.078693664	1.366008130
C	0.000000000	0.000000000	0.612850130
O	-1.145797034	0.078693664	1.366008130
C	0.000000000	0.000000000	-0.718624870
C	-1.255219462	0.244533057	-1.505942370
H	1.012874917	-0.737290125	2.061427630
H	-1.012874917	0.737290125	2.061427630
H	-2.104214586	0.469695653	-0.864420370
H	-1.510771363	-0.630789217	-2.112497370
H	-1.115994359	1.077461722	-2.203325370
C	1.255219462	-0.244533057	-1.505942370
H	1.115994359	-1.077461722	-2.203325370
H	1.510771363	0.630789217	-2.112497370
H	2.104214586	-0.469695653	-0.864420370

E (DLPNO-CCSD(T)) = –307.185573

ZPVE (B3LYP) = 0.116715

2tt: Enol *trans*, *trans* (C_2)

O	1.083337877	0.118302648	1.438962043
C	0.000000000	0.000000000	0.631802043
O	-1.083337877	-0.118302648	1,.438962043
C	0.000000000	0.000000000	-0.709538957
C	-1.270548116	-0.056891521	-1.515004457
H	1.842631870	0.382452339	0.909460543
H	-1.842631870	-0.382452339	0.909460543
H	-1.263390461	0.708390396	-2.298029457
H	-2.173104387	0.124970314	-0.926557457
H	-1.396130499	-1.021446915	-2.020540457
C	1.270548116	0.056891521	-1.515004457
H	2.173104387	-0.124970314	-0.926557457
H	1.263390461	-0.708390396	-2.298029457
H	1.396130499	1.021446915	-2.020540457

E (DLPNO-CCSD(T)) = –307.183604

ZPVE (B3LYP) = 0.116663 kcal mol^{-1}

4: Ketene (C_{2v})

C	0.000000000	1.303947000	1.207449284
C	0.000000000	0.000000000	0.444917784
C	0.000000000	-1.303947000	1.207449284
C	0.000000000	0.000000000	-0.864729216
O	0.000000000	0.000000000	-2.031687216
H	0.882896500	1.374534250	1.850274534
H	-0.882896500	1.374534250	1.850274534
H	0.000000000	2.168505001	0.544041283
H	0.882896500	-1.374534250	1.850274534
H	0.000000000	-2.168505001	0.544041283
H	-0.882896500	-1.374534250	1.850274534

E (DLPNO-CCSD(T)) = –307.172230

ZPVE (B3LYP) = 0.089243

5: Propene (C_s)

C	-1.172646274	0.483148701	0.000000000
C	-0.006803447	-0.455238205	0.000000000
C	1.270016044	-0.093251064	0.000000000
H	-0.848607147	1.524897648	0.000000000
H	-1.806445605	0.321390543	0.877046000
H	-1.806445605	0.321390543	-0.877046000
H	-0.249314836	-1.515201687	0.000000000
H	1.564688620	0.950639136	0.000000000
H	2.067766839	-0.825117202	0.000000000

E (DLPNO-CCSD(T)) = –117.681381

ZPVE (B3LYP) = 0.075155

6: Dimethylcarbene (C_s)

C	-1.221051000	0.155619000	0.020527000
C	0.000001000	-0.656296000	-0.000001000
C	1.221050000	0.155617000	-0.020527000
H	-1.459861000	0.153089000	-1.061157000
H	-1.133138000	1.212050000	0.322672000
H	-2.073059000	-0.329963000	0.498358000
H	1.133141000	1.212047000	-0.322684000
H	2.073069000	-0.329962000	-0.498340000
H	1.459842000	0.153102000	1.061162000

E (DLPNO-CCSD(T)) = –117.570761

ZPVE (B3LYP) = 0.075155

TS1: Enol rotation **2ct** – **2tt** (C_1)

O	-1.493194000	1.136410000	-0.030097000
C	-0.710174000	0.018056000	-0.007454000
O	-1.508419000	-1.090671000	-0.051178000
C	0.626602000	-0.016539000	0.005252000
C	1.381235000	-1.315423000	-0.051813000
H	-0.941485000	1.911038000	-0.185962000
H	-1.572060000	-1.488623000	0.824003000

H	0.721355000	-2.163281000	-0.223998000
H	1.939792000	-1.495939000	0.873842000
H	2.118911000	-1.296880000	-0.861136000
C	1.469861000	1.229311000	0.054461000
H	2.266371000	1.121805000	0.796857000
H	1.960331000	1.424388000	-0.905686000
H	0.914542000	2.129145000	0.329596000

E (DLPNO-CCSD(T)) = –307.181066

ZPVE (B3LYP) = 0.115846

TS2: Enol rotation **2ct** – **2cc** (C_1)

O	-1.415811000	1.160018000	-0.086153000
C	-0.684254000	-0.000001000	0.007492000
O	-1.415810000	-1.160020000	-0.086152000
C	0.648416000	-0.000001000	0.026197000
C	1.437315000	-1.277619000	0.009714000
H	-2.167573000	1.140555000	0.518393000
H	-2.167586000	-1.140546000	0.518377000
H	0.798698000	-2.156231000	0.061748000
H	2.137551000	-1.306913000	0.851026000
H	2.041455000	-1.350123000	-0.901289000
C	1.437312000	1.277621000	0.009714000
H	2.137577000	1.306898000	0.851001000
H	2.041421000	1.350146000	-0.901309000
H	0.798694000	2.156229000	0.061788000

E (DLPNO-CCSD(T)) = –307.179609

ZPVE (B3LYP) = 0.115682

TS3: Enol **2tt** – isobutyric acid **3t** (C_1)

O	-1.415726000	-1.037487000	-0.511741000
C	-0.799641000	-0.033657000	-0.008913000
O	-1.526312000	0.933390000	0.528953000
C	0.607322000	-0.079870000	-0.258166000
C	1.348678000	1.232486000	-0.399359000
H	-0.287064000	-1.077002000	-1.046093000
H	-0.944120000	1.567697000	0.970179000
H	0.788541000	1.967570000	-0.981802000

H	2.297177000	1.073861000	-0.916331000
H	1.604214000	1.688059000	0.571709000
C	1.388415000	-1.142814000	0.518578000
H	0.803637000	-2.047023000	0.689885000
H	2.284554000	-1.430083000	-0.035085000
H	1.720719000	-0.767178000	1.497006000

E (DLPNO-CCSD(T)) = –307.102606

ZPVE (B3LYP) = 0.112009

TS4: Enol **2ct** – isobutyric acid **3c** (C_1)

O	1.367934000	1.071542000	-0.488095000
C	0.772672000	0.036688000	0.011466000
O	1.485670000	-0.947412000	0.534180000
C	-0.623709000	0.032075000	-0.219795000
C	-1.346357000	-1.290439000	-0.334846000
H	0.234045000	1.033352000	-1.038384000
H	2.424169000	-0.788926000	0.346574000
H	-1.672873000	-1.671697000	0.641798000
H	-0.727959000	-2.057975000	-0.801832000
H	-2.244046000	-1.173168000	-0.946405000
C	-1.442922000	1.129542000	0.462144000
H	-0.873834000	2.047196000	0.612463000
H	-2.320038000	1.377455000	-0.139057000
H	-1.806396000	0.793527000	1.442345000

E (DLPNO-CCSD(T)) = –307.105018

ZPVE (B3LYP) = 0.112018

TS5: Enol **2ct** – ketene (**4**) – H_2O elimination (C_1)

O	1.687909000	0.895317000	-0.052184000
C	0.577958000	-0.276418000	-0.017554000
O	1.408743000	-1.242808000	-0.033075000
C	-0.712885000	0.028075000	0.000334000
C	-1.700044000	-1.110965000	0.042176000
H	1.721343000	1.354225000	0.801659000
H	2.200653000	-0.150406000	-0.017299000
H	-2.349263000	-1.032453000	0.920581000
H	-1.199001000	-2.077252000	0.076448000

H	-2.348707000	-1.095923000	-0.839986000
C	-1.256797000	1.425978000	-0.040129000
H	-1.944322000	1.547964000	-0.883796000
H	-1.829125000	1.657929000	0.865121000
H	-0.474195000	2.175822000	-0.149614000

E (DLPNO-CCSD(T)) = –307.111571

ZPVE (B3LYP) = 0.111146

TS6: Ketene [1,2]H-shift (C_1)

C	-0.980759000	1.338604000	0.073653000
C	-0.377696000	-0.045571000	-0.148201000
C	-1.281453000	-1.264800000	0.215773000
C	0.978031000	-0.065052000	-0.103101000
O	2.125210000	-0.078988000	0.077063000
H	-1.233522000	1.443232000	1.129553000
H	-1.900543000	1.419494000	-0.505347000
H	-0.312552000	2.143782000	-0.235047000
H	-0.754709000	-2.211757000	0.290383000
H	-0.659043000	-0.636289000	-1.111100000
H	-2.170047000	-1.305647000	-0.413690000

E (DLPNO-CCSD(T)) = –230.688644

ZPVE (B3LYP) = 0.082933

TS7: Singlet Carbene [1,2]H-shift (C_1)

C	1.214734000	-0.205807000	-0.037254000
C	0.081142000	0.591727000	-0.116676000
C	-1.217209000	-0.139433000	0.016036000
H	2.202590000	0.227026000	-0.185592000
H	1.192317000	-1.305363000	0.035195000
H	0.877623000	0.505813000	0.953447000
H	-1.147309000	-1.213400000	0.253269000
H	-1.712071000	-0.042422000	-0.956458000
H	-1.885155000	0.349424000	0.727501000

E (DLPNO-CCSD(T)) = –117.558509

ZPVE (B3LYP) = 0.073481
